# Biobanking in the digital pathology era

**DOI:** 10.32604/or.2022.024892

**Published:** 2022-08-31

**Authors:** GIUSEPPINA BONIZZI, LORENZO ZATTONI, NICOLA FUSCO

**Affiliations:** Biobank for Translational and Digital Medicine Unit, Division of Pathology, IEO, European Institute of Oncology IRCCS, University of Milan, Milan, 20141, Italy

**Keywords:** Biobank, Pathology, Digital pathology, Biomarkers, Translational research

## Abstract

Digital Pathology is becoming more and more important to achieve the goal of precision medicine. Advances in whole-slide imaging, software integration, and the accessibility of storage solutions have changed the pathologists’ clinical practice, not only in terms of laboratory workflow but also for diagnosis and biomarkers analysis. In parallel with the pathology setting advancement, translational medicine is approaching the unprecedented opportunities unrevealed by artificial intelligence (AI). Indeed, the increased usage of biobanks’ datasets in research provided new challenges for AI applications, such as advanced algorithms, and computer-aided techniques. In this scenario, machine learning-based approaches are being propose in order to improve biobanks from biospecimens collection repositories to computational datasets. To date, evidence on how to implement digital biobanks in translational medicine is still lacking. This viewpoint article summarizes the currently available literature that supports the biobanks’ role in the digital pathology era, and to provide possible practical applications of digital biobanks.

## Introduction

In the last decades, biobanking has emerged as one of the most important tasks in cancer research, by providing samples and data collection for appropriate analysis and experiments [1–3]. In literature, a growing number of studies have been carried out using human biological specimens and related data processed by biobanks [4–8]. The analysis of diverse sets of data stored in biobanks from a variety of sources involving bioinformatics and omics sciences is extremely useful in cancer research [2,9,10]. Biobanks can provide datasets containing patients’ lifestyle and disease information matched with different types of biological specimens, including tissue samples and biofluids, and annotated pathological data [11–15]. However, biobanks face limits due to data gaining, storage, and practice [16–19]. Despite the possibility to collect a high number of data it is important to optimize all this information. These difficulties are intrinsic to biobank data acquisition, but machine learning technologies can help overcome the related issues [20–22]. In this context, as the practice of precision medicine is taking advantage of artificial intelligence techniques (AI), biobanks are starting to evolve from biospecimen collection repositories to integrated computational datasets [23]. Thus, AI and machine learning applications may significantly impact biobanking in cancer research [24,25]. In this Viewpoint article, we discuss the biobanks’ role in the digital pathology era, along with future perspectives for AI and machine learning applications in biobanking.

### What Is a Biobank?

**Figure 1:**
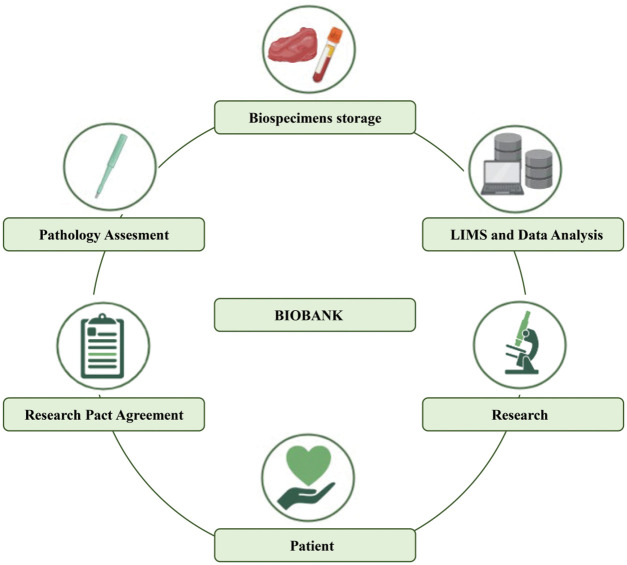
Traditional biobank workflow.

Biobanks represent biorepositories of various types of biological samples and all associated data, aimed not only at storing a wide variety of data for sample preservation but also at processing them to provide the armamentarium for translational research and clinical studies [3,26]. Samples that can be collected by biobanks include tissues (normal and/or abnormal), biofluids (e.g., blood, serum, plasma, urine, saliva, liquor, effusion, bone marrow fluid portion, sperm, cord blood), stool, cells, peripheral blood cells (PBCs), and nucleotides (e.g., DNA, RNA, miRNA). All these biospecimens are integrated into datasets containing the associated data (i.e., clinicopathologic, genetic, and personal data). For this purpose, it is crucial to complement samples with up-to-date available related data. To proof the quality of the samples and procedures, biobanks need appropriate accreditations (e.g., ISO 15189 and ISO 17025), and an operational quality management system to guarantee consistent quality and results. Moreover, biobanks periodically implement international guidelines (e.g., U.S. National Cancer Institute) with standard operating procedures (SOPs) for maintaining high-quality processes [27–29]. In the first place, biobanks were established to preserve biospecimens over time [30]. To this end, the samples are usually stored in cryogenic facilities, including either specific refrigerators or warehouses. In addition, biobanks may collect matched formalin-fixed paraffin-embedded (FFPE) tissues [31]. Of note, tissues stored in biobanks may be originated from surgical specimens’ leftover, minor surgery ultrasound-guided biopsies [32]. All collected biological samples and related data follow a well-defined standard operating procedure and are always obtained after the patient sign the research pact agreement [28]. Taken together, each component in the biobank workflow is important to guarantee the quality of the sample, and its reliability in data analysis (Fig. 1). A modern biobank should be able to interact with different types of interlocutors, including research groups, clinical units, political institutions, biotech companies, and the pharma industry. Each time a project requires a biobank to support the scientific objectives, it is essential to provide well-preserved samples and data and meet prescribed requirements. Data must be safe, accessible, and traceable to manage simultaneously different projects. Further, another critical aspect of biobanking management is to have an integrated laboratory information management system (LIMS) software that can automatically integrate all clinical records [33–35]. To secure this integration, it is critical to obtain the scientific participation agreement of all patients for legal and ethical requirements [28].

### The Digital Pathology Era

Advancements in AI applications have led to a transformed practice in the healthcare system [24,36]. Nowadays, the healthcare system is facing a biomedical big data issue, with an extensive amount of data available to analyze that is difficult to be manually processed [37]. Digital pathology represents a new era for pathology practices. This technique involves a full scanning of glass histological slide, into a digitized image data called whole slide image (WSI). This file can be stored in a database and easily available to any researcher from a computer screen. In this approach, due to the advances in AI, the use of digital microscopy is increasing for either educational or research purposes [38–42]. Moreover, WSI can be utilized in image analyses by integrating deep learning models for identification and segmentation [43,44]. Taken together, digital pathology is significantly and rapidly changing the pathology workflow [45,46].

### Evolving Towards Digital Biobanks: Opportunities and Critical Aspects

**Figure 2:**
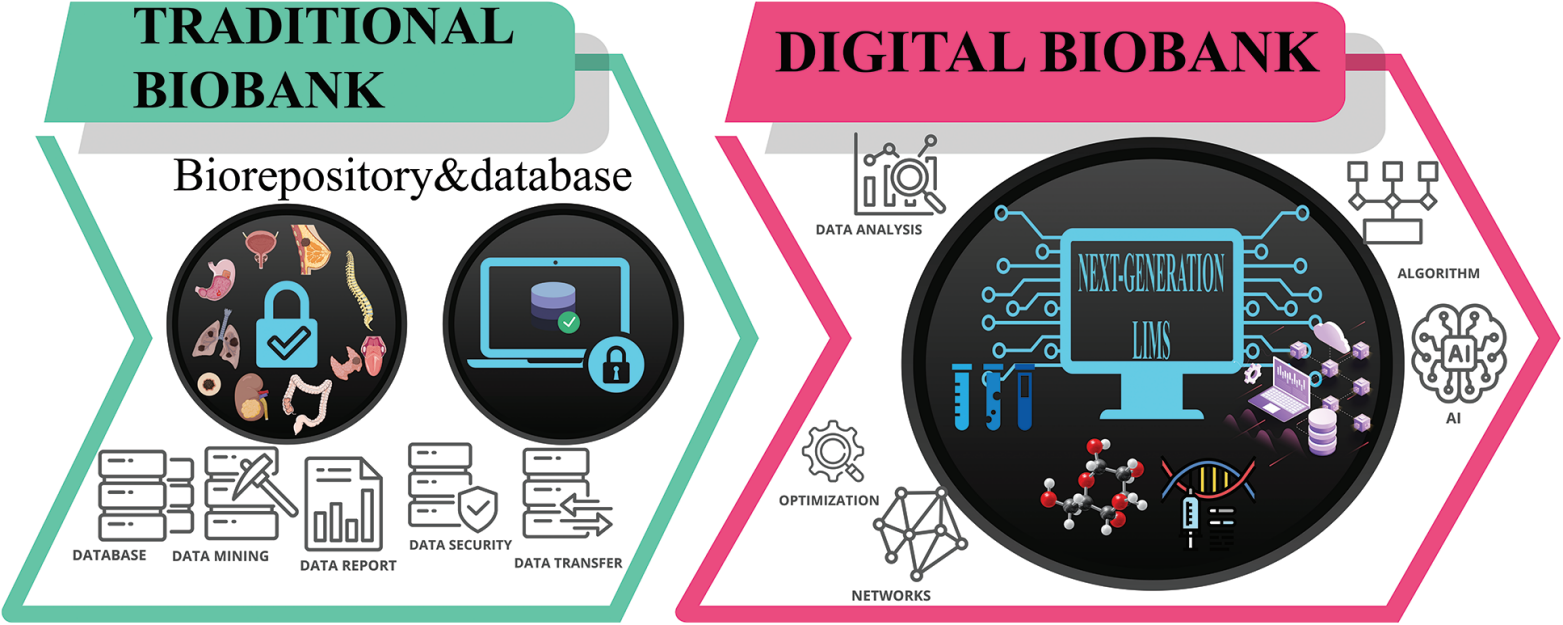
Model for digital biobank.

Given the central role of biobanks in modern research for data acquisition and data-sharing, the integration of different types of information should be investigated. This problem may be addressed with the use of AI and machine learning models [47]. However, there are too few examples of digitalized biobanks worldwide, making this progress in its early journey stage [17,48]. Still, scientists are starting training algorithms on datasets for meaningful findings [49,50]. Specifically, machine learning techniques have shown novel interesting applications in processing data for predictive models, such as detecting early signals of cancers [51]. For this, new staffing models need to be implemented with widely different expertise than the traditional biobank pathology technician. In this respect, a multidisciplinary team of dedicated and trained professionals would be required in order to embrace the challenges provided by the evolution towards the concept of digital biobank. Amon these, pathologists, molecular oncologists, bioinformaticians, bioengineers, programmers and IT specialists are needed to work in close collaborations with biologists, biotechnologists, and laboratory technicians. The recent increase in data collection has pointed out the need to overcome manual handling to the use of machine learning models to run analyses. In this scenario, AI techniques, and more specifically deep learning models, can bring several advantages. Therefore, looking back at the literature, studies underlined the impact of a computational approach to improve patient safety [52], healthcare quality [53], and reduce costs [54]. More in detail, biobanks can report significant improvement in integrating data derived from samples (e.g., -omics data) with medical records, or other types (e.g., pathological data) [48]. Moreover, developing an available online database of whole slide images for biobank specimens might increase the sample’s accessibility to a broader audience of researchers [55]. Interestingly, datasets would become visually inspectable, leading to benefits in biospecimens’ preselection, consultation, and reuse. In this context, incorporating digital pathology into biobank standard operating procedures might minimize the inspector variability associated with routine pathologic evaluations in biorepositories [56]. On the other hand, several critical aspects have to be considered to implement AI techniques. Firstly, each piece of data has to be fully digitalized to allow storage and processing. Besides data still needs to be consented to by patients, deidentified, and already managed in a way understandable for deep learning models [48]. Deep learning models can bridge the gap between biobanks and evolving concept of AI and translational research. However, moving towards a purely digital biobank would represent a quantum shift for biobanks to actually be the research hub. Therefore, the main question is: Do Biobanks have the resources to do this? It is a matter of fact that cutting-edge digital technologies require human expertise, time to be implemented, but most of all the infrastructures [57]. The “2.0 staff” required for digital biobanks should work in synergy with machine learning algorithms, providing dedicated training to work not only with biospecimens and data, but also with algorithms. In this respect, the integration of existing software would be germane to provide researchers, patients, and stakeholders with accessible and organized data (Fig. 2).

## Conclusion

Owing to the unprecedented opportunities provided in the digital pathology era by big data collection and artificial intelligence, every laboratory will have access to an increasing amount of data. In this new scenario, biobanks will inevitably represent a bridge between biological and digital data [23,58]. Many benefits are achievable in this process. A digital biobank allows the integration of different expertise from remote, making possible teleconsultation [59]. Indeed, when addressing cancer research, a collaboration of a multidisciplinary board becomes important for the discussion and interpretation of each specific project [60]. In addition, biobanks continuously demand improved efficiency in storage and sustainability [61,62]. To accomplish these requirements, next-generation biobanking will face the green hospital revolution, as a response to the growing concern over resource wasting and environmental damage [63,64]. Digital biobanks will decrease resource usage with the dematerialization of data and samples and reduce energy use by digitalizing data storage and tracking, still providing patients with the best service. Finally, despite the current efforts in biobanking standardization and harmonization for data collection, we believe that future achievements lie in adding digital pathology and artificial intelligence technology to the existing repositories. To acknowledge biobanks as a fundamental part of scientific research, only AI integration in large datasets may be the requirement for improved biobanks’ standard operating procedures in the digital pathology era.

## Data Availability

Not applicable.
